# SARS-CoV-2 Genomic Variation in Space and Time in Hospitalized Patients in Philadelphia

**DOI:** 10.1128/mBio.03456-20

**Published:** 2021-01-19

**Authors:** John Everett, Pascha Hokama, Aoife M. Roche, Shantan Reddy, Young Hwang, Lyanna Kessler, Abigail Glascock, Yize Li, Jillian N. Whelan, Susan R. Weiss, Scott Sherrill-Mix, Kevin McCormick, Samantha A. Whiteside, Jevon Graham-Wooten, Layla A. Khatib, Ayannah S. Fitzgerald, Ronald G. Collman, Frederic Bushman

**Affiliations:** a Department of Microbiology, Perelman School of Medicine, University of Pennsylvania, Philadelphia, Pennsylvania, USA; b Pulmonary, Allergy and Critical Care Division, Department of Medicine; University of Pennsylvania Perelman School of Medicine, Philadelphia, Pennsylvania, USA; Washington University School of Medicine in St. Louis

**Keywords:** SARS-CoV-2, COVID-19, coronavirus, genome sequencing, Philadelphia

## Abstract

The severe acute respiratory coronavirus 2 (SARS-CoV-2) is the cause of the global outbreak of COVID-19. The epidemic accelerated in Philadelphia, PA, in the spring of 2020, with the city experiencing a first peak of infections on 15 April, followed by a decline through midsummer. Here, we investigate spread of the epidemic in the first wave in Philadelphia using full-genome sequencing of 52 SARS-CoV-2 samples obtained from 27 hospitalized patients collected between 30 March and 17 July 2020. Sequences most commonly resembled lineages circulating at earlier times in New York, suggesting transmission primarily from this location, though a minority of Philadelphia genomes matched sequences from other sites, suggesting additional introductions. Multiple genomes showed even closer matches to other Philadelphia isolates, suggestive of ongoing transmission within Philadelphia. We found that all of our isolates contained the D614G substitution in the viral spike and belong to lineages variously designated B.1, Nextstrain clade 20A or 20C, and GISAID clade G or GH. There were no viral sequence polymorphisms detectably associated with disease outcome. For some patients, genome sequences were determined longitudinally or concurrently from multiple body sites. In both cases, some comparisons showed reproducible polymorphisms, suggesting initial seeding with multiple variants and/or accumulation of polymorphisms after infection. These results thus provide data on the sources of SARS-CoV-2 infection in Philadelphia and begin to explore the dynamics within hospitalized patients.

## Abstract (precis)

Understanding how SARS-CoV-2 spreads globally and within infected individuals is critical to the development of mitigation strategies. We found that most lineages in Philadelphia had resembled sequences from New York, suggesting infection primarily but not exclusively from this location.

## INTRODUCTION

The disease COVID-19 is caused by infection with the betacoronavirus SARS-CoV-2 (1). As with other coronaviruses, transmission typically takes place via droplets and aerosols or by contact with contaminated surfaces (2–4). SARS-CoV-2 was identified first in China in December 2019 and later in 2020 in most countries. The World Health Organization declared COVID-19 a global pandemic on 11 March 2020.

In the United States, SARS-CoV-2 infection was first recognized in the state of Washington in January 2020. By March 2020, outbreaks had been detected in all 50 states (5). High infection rates were detected in New York City in the spring of 2020, with the first case identified on 29 February 2020 (6).

**FIG 1:**
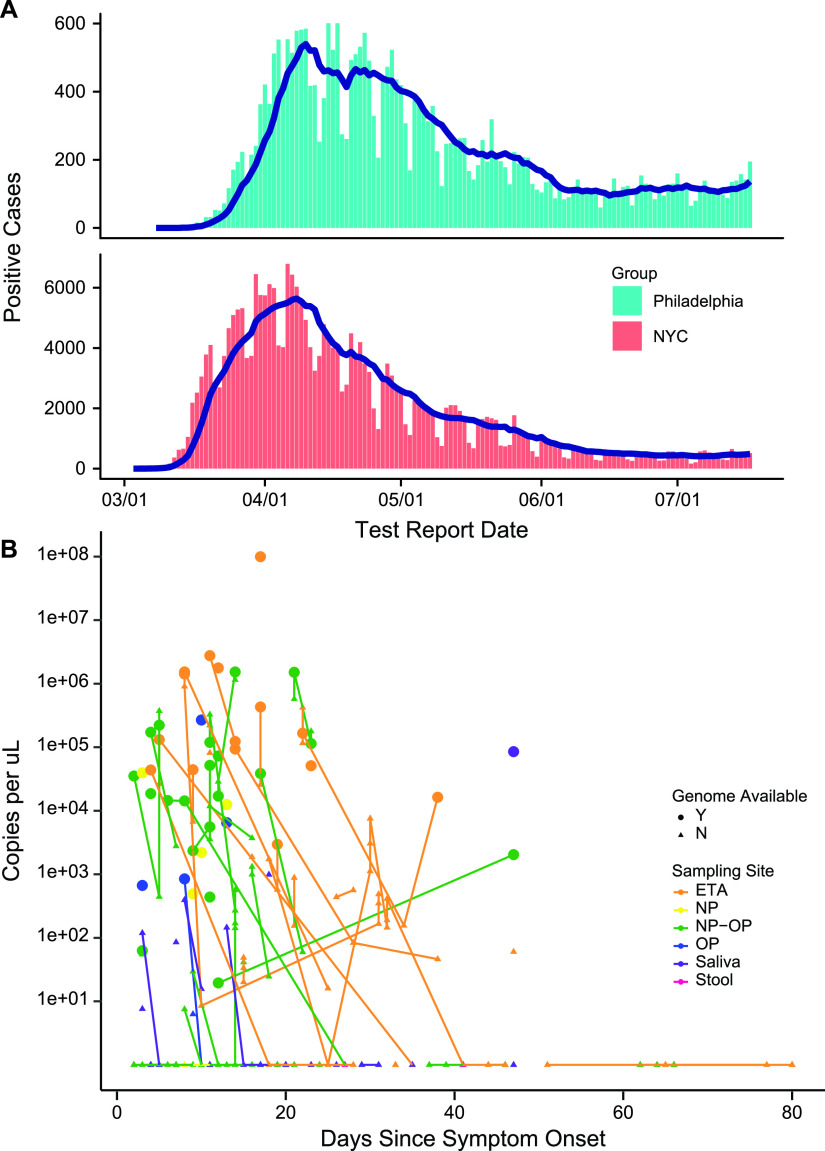
The first wave of the COVID-19 epidemic in Philadelphia. (A) Number of positive cases recorded per day in Philadelphia and New York City from March to July 2020. Data are provided by the city of New York (https://www1.nyc.gov/site/doh/covid/covid-19-data-testing.page) and the city of Philadelphia (https://www.phila.gov/programs/coronavirus-disease-2019-covid-19/testing-and-data/). (B) Levels of SARS-CoV-2 RNA detected in patient samples using RT-qPCR. The *x* axis shows days after symptom onset, and the *y* axis shows the number of viral RNA copies in samples after RNA purification. Colors show sample type. Colored lines connect samples of the same type obtained longitudinally from within each patient. All samples available are shown for each subject who provided at least one high-quality genome sequence of that sample type. ETA, endotracheal aspirate; NP, nasopharyngeal; OP, oropharyngeal.

Here, we investigate samples from the later wave of infection in the city of Philadelphia, PA, taking advantage of viral whole-genome sequencing. The first case of COVID-19 was detected in Philadelphia on 8 March 2020. The epidemic spread rapidly, reaching a first peak of 601 newly diagnosed cases on 17 April 2020 and waned during the remainder of our sampling period, which closed 17 July 2020 (Fig. 1A).

A variety of polymorphisms have been described in the SARS-CoV-2 genome, which provide a means of tracking infections (5–11) and also a window on viral biology. One notable substitution encodes the spike protein (S) variant D614G. The viral spike protein is present on the viral surface and is responsible for binding to the ACE2 host cell receptor and directing membrane fusion and viral entry. The D614G substitution has been proposed to promote infection of human cells, and this variant has spread globally at the expense of other genotypes (12–15). The D614G variant in recently isolated genomes often cooccurs with a mutation encoding P314L in the virus-encoded RNA-dependent RNA polymerase (RdRp) located on ORF1b.

Here, we report the sequence of 52 high-quality genomes from 27 subjects in Philadelphia, allowing investigation of the origins of the local epidemic and assessment of viral variation in patients. All genomes contained the D614G spike substitution and the P314L RdRp substitution. Several nomenclatures have been proposed for this SARS-CoV-2 lineage—these include lineage B.1, Nextstrain clade 20A or 20C, and GISAID clade G or GH; an older designation is the A2a clade (16, 17). Comparison of viral genomes from Philadelphia to sequences from other locations showed that those from New York were commonly the most similar, suggesting a source of the local epidemic. A minority of cases showed nearest neighbors from other sites, suggesting additional introductions from other locations. Comparison of genomes from within Philadelphia showed that many had even better matches to local isolates, suggestive of community spread. We also investigate viral genome variants present at different body sites in patients and longitudinally over time, revealing reproducible polymorphisms in some cases. Lastly, we did not find any strong association of viral genome variation with patient outcomes, as in previous work (18), indicating that subject-specific factors rather than virus-specific factors likely dominate clinical course.

## RESULTS

### The COVID-19 epidemic in Philadelphia.

Whole-genome sequences were obtained from 27 patients hospitalized at the Hospital of the University of Pennsylvania in Philadelphia (Table 1; see also Table S1 in the supplemental material). The sampling period began on 30 March, 22 days after the first detection of SARS-CoV-2 in Philadelphia, extended through the peak daily case number of 601 on 15 April, and was closed after daily cases fell to 195 on 17 July 2020 (Fig. 1A). Of the 27 patients, 12 were female and 15 were male, 20 were Black, five were white, one was Asian, and one identified as other. The median age was 64 years (range 28 to 90), and all but one had at least one underlying major organ system comorbidity (Table 1). Twenty-one subjects received corticosteroids, 12 received hydroxychloroquine, and five received remdesivir. Seventy-eight percent required intubation. Nineteen recovered and left the hospital, and eight died. There were no significant differences between surviving and nonsurviving patients in age, gender, race, underlying comorbidities, or treatment, although the numbers compared were modest.

**TABLE 1:** Characteristics of the study participants^*a*^

	All (*n* = 27)	Nonsurvivors (*n* = 8)	Survivors (*n* = 19)
Age, median (range)	64 (28–90)	66 (46–85)	62 (28–90)
Male/female	15/12	4/4	11/8
Race			
Black	20	5	15
White	5	2	3
Asian	1	1	0
Other	1	0	1
Hispanic/Latinx	0	0	0
Major comorbidities			
Diabetes	15	5	10
Hypertension	24	8	16
CAD/CVD	12	5	7
Cancer	4	3	1
HIV	2	1	1
Organ transplant	1	0	1
Chronic lung disease	12	3	9
Renal disease (≥stage 4)	8	2	6
None	1	0	1
BMI, median (range)	31.1 (17–48)	27.5 (17–46)	31.1 (17–48)
Mod/severe obesity (BMI >35)	8	2	6
Treatment			
Corticosteroids	21	7	14
Hydroxychloroquine	12	3	9
Remdesivir	5	2	3
Max WHO score, median (range)		10	8 (4–9)
Days from hospitalization to discharge/death, median (range)	24 (3–61)	22.5 (10–39)	32 (4–63)

a Abbreviations: CAD, coronary artery disease; CVD, cardiovascular disease; BMI, body mass index; Mod, moderate.

10.1128/mBio.03456-20.1 TABLE S1 Table of COVID-19 patients studied. Download Table S1, XLSX file, 0.01 MB. Copyright © 2021 Everett et al. 2021 Everett et al. This content is distributed under the terms of the Creative Commons Attribution 4.0 International license.

High-quality complete genome sequences (Table S2) were obtained from 52 samples. Samples yielding high-quality genome sequences included nasopharyngeal (NP) swabs *n* = 6), oropharyngeal (OP) swabs (*n* = 8), pooled NP+OP swabs (*n* = 21), saliva (*n* = 1), and endotracheal aspirates (ETA) of lung secretions from intubated patients (*n* = 16). In nine cases, viral genomes were expanded in cell culture prior to sequence acquisition. Sequences were also obtained from two samples of the reference isolate USA-WA1-2020, which was isolated in Seattle, WA, from the first patient identified in the United States on 20 January 2020.

10.1128/mBio.03456-20.2 TABLE S2 Table of genomes with metadata and accession numbers. TCE indicates tissue culture expanded. Download Table S2, XLSX file, 0.01 MB. Copyright © 2021 Everett et al. 2021 Everett et al. This content is distributed under the terms of the Creative Commons Attribution 4.0 International license.

Viral genome copy numbers were assayed in each sample (Fig. 1B). Viral RNA copies per sample typically fell with time after symptom onset, as has been reported previously in many studies. A minority of patients had prolonged RNA detection, allowing analysis of genome sequences over 1 month of viral persistence in the infected subjects.

### Whole-genome sequence analysis of SARS-CoV-2.

Genomes were analyzed by reverse transcription of the viral RNA to make a cDNA copy, PCR amplification of genome segments, Nextera library preparation, and Illumina sequencing. Initially, we devised a protocol based on amplifying the viral genome in six segments. We successfully sequenced several SARS-CoV-2 isolates but noticed that high concentrations of viral RNA were required for efficient amplification. We thus substituted the ARTIC primer set, which amplifies the SARS-CoV-2 genome as 98 shorter amplicons (19). We found that this protocol yielded complete genome sequences more efficiently than the six-amplicon protocol, likely because of greater PCR efficiency with shorter amplicons. We note that our sequencing approach yields the average base at each position in the population—in the interest of high throughput, no effort was made to isolate single genomes prior to sequence acquisition.

An analytical pipeline was devised based on the POLAR protocol for sequence assembly and characterization (19). Sequence reads were aligned to the USA-WA1-2020 reference genome, and variants were identified (Table S3). To be accepted for analysis, genome sequences were required to have least 95% coverage of the USA-WA1-2020 reference (20) and a minimum read depth of 5 reads per position. Results from two subjects were excluded due to failure to meet our sample quality control standards.

10.1128/mBio.03456-20.3 TABLE S3 Table of polymorphisms present in isolates. Download Table S3, XLSX file, 0.01 MB. Copyright © 2021 Everett et al. 2021 Everett et al. This content is distributed under the terms of the Creative Commons Attribution 4.0 International license.

### Investigating the origin of the epidemic in Philadelphia.

**FIG 2:**
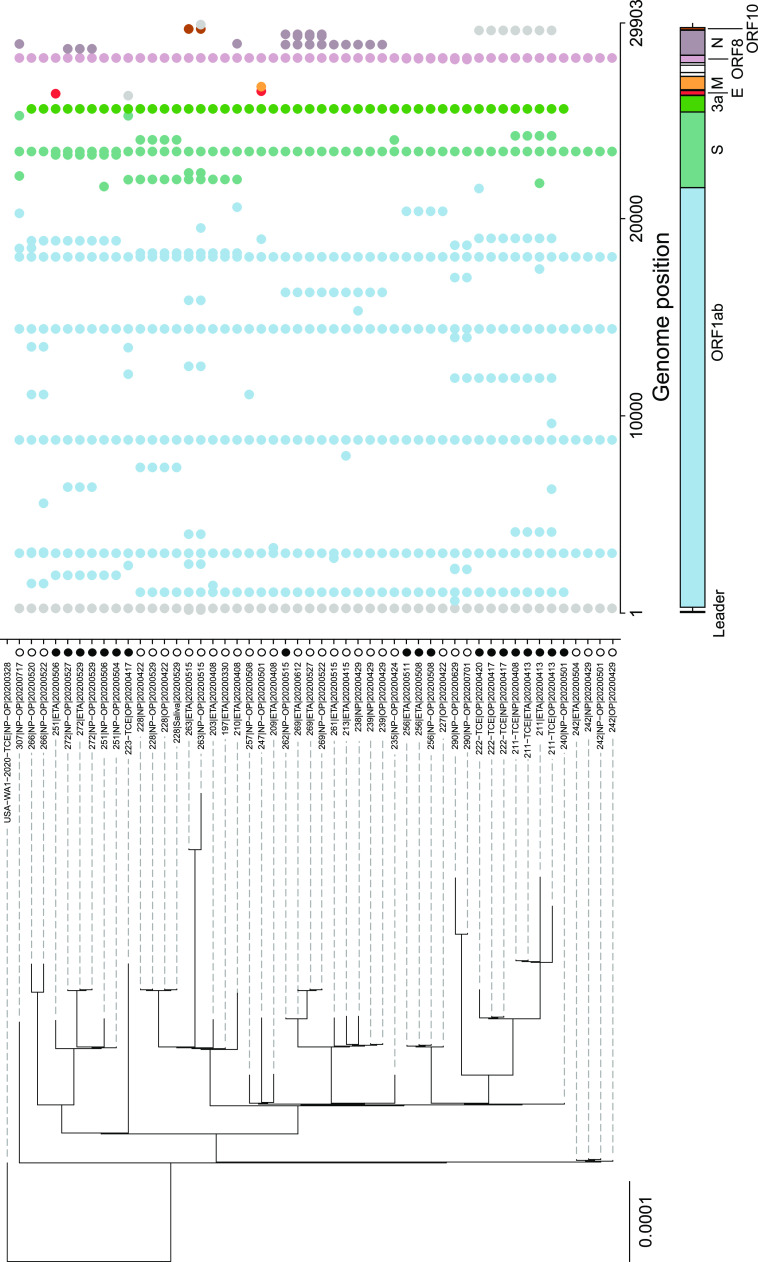
Sequence polymorphisms in SARS-CoV-2 isolates from Philadelphia. Polymorphisms are shown relative to the USA-WA1-2020 reference isolate. A tree generated using hierarchical clustering (UPGMA method) is shown to the left; a map of the SARS-CoV-2 genome with sequence polymorphisms indicated is shown to the right. Each row indicates a single genome, and each column indicates a nucleotide position. The dots indicate sequence polymorphisms; each is color coded by the SARS-CoV-2 gene in which it was found (code at bottom). Patient outcomes are encoded by the column of symbols between the tree and the map of polymorphisms, coded as follows: open circle, survived infection; filled circle, fatal outcome. The sequence of the USA-WA1-2020 isolate was verified twice independently by sequencing.

Polymorphisms were identified in viral genomes isolated in Philadelphia by comparison to the USA-WA1-2020 reference isolate (20). Sites of polymorphisms were cataloged, and genomes were arranged on a phylogenetic tree (Fig. 2 and Tables S2 and S3). Samples from the same subjects clustered together for 26 of the 27 subjects. In the exceptional case, subject 211, the comparisons showing differences involved genomes that were sequenced directly versus isolates that were expanded in tissue culture prior to sequencing and so may be a consequence of the expansion procedure; this is discussed further below.

All genomes from Philadelphia were found to encode the D614G spike polymorphism suggested to promote efficient spread in humans (13, 21, 22). Philadelphia sequences also all encoded P314L in the virus-encoded RdRp (ORF1b), marking them as lineage B.1, Nextstrain clade 20A or 20C, GISAID clade G or GH, and clade A2a (16, 17). All genomes contained further polymorphisms distinguishing them from the USA-WA1-2020 reference isolate (Fig. 2).

**FIG 3:**
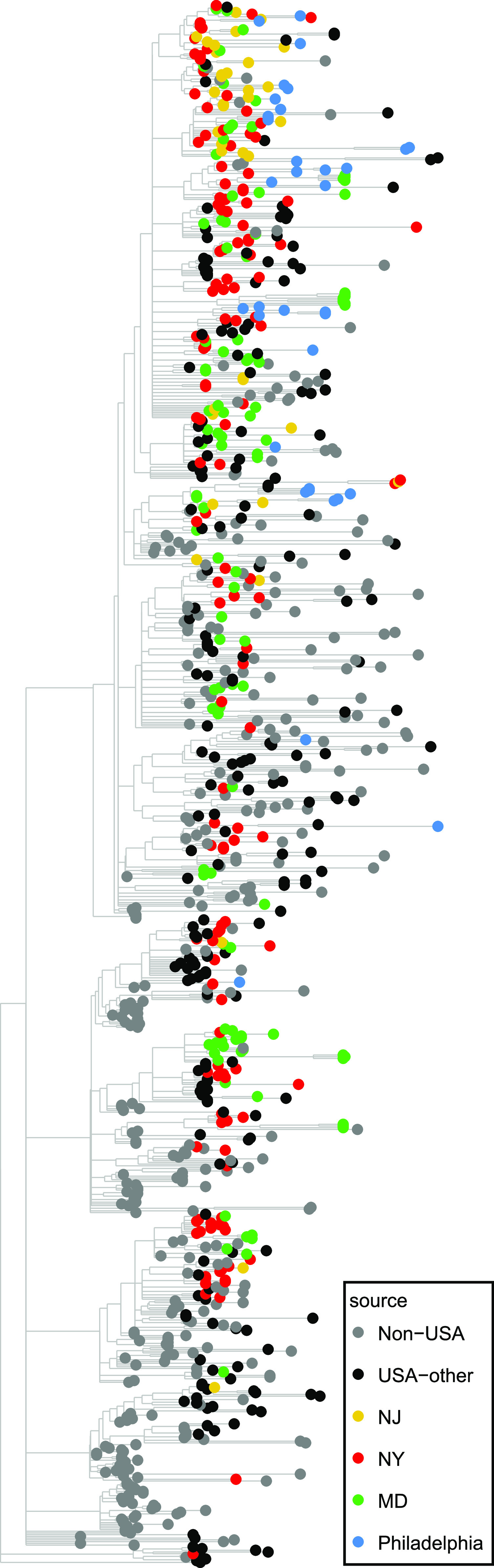
SARS-CoV-2 isolates in the global context. Isolates are indicated by the global site of origin. The phylogenetic tree shows isolates from 30 March 2020 and later, including the lineages sampled here. Phylogenetic trees were generated via the IQ-TREE algorithm (48). The geographic origin of selected sequence isolates is shown by the color code.

SARS-CoV-2 genomes from Philadelphia were compared to global sequences at several time points in the epidemic. The lineage B.1/Nextstrain clade 20A or 20C/GISAID clade G or GH/A2a (16, 17) variants were circulating in New York (6) prior to expansion of the epidemic in Philadelphia. Figure 3 shows clustering of strains from Philadelphia with New York and global strains. To investigate the geographical origin of the epidemic in Philadelphia more carefully, each genome was aligned to database genomes and neighbors with the lowest edit distances were recorded (Table S4). In this analysis, only global isolates were selected for comparison that were reported prior to the date of symptom onset for each patient queried. Comparisons commonly showed nearest neighbors at multiple locations; in the text below, the majority location is emphasized.

10.1128/mBio.03456-20.4 TABLE S4 Table summarizing nearest neighbor analysis of SARS-CoV-2 isolates from Philadelphia. Download Table S4, XLSX file, 0.01 MB. Copyright © 2021 Everett et al. 2021 Everett et al. This content is distributed under the terms of the Creative Commons Attribution 4.0 International license.

For 22 of the 27 subjects, the most frequently identified closest-matched database genomes were from subjects in New York. Other Philadelphia genomes showed most frequent best alignments to sequences from Massachusetts (subject 223), Sweden (subject 242), California (subjects 263 and 307), and New Jersey (subject 266). In these comparisons, mismatches between Philadelphia and global sequences ranged from 0 to 10 substitutions (mean = 2.3). None of the subjects with sequences linked to Sweden, Massachusetts, or California had known direct contact with these locations prior to illness, suggesting community spread as the proximal source.

To assess the contribution of local circulation of lineages, our genome sequences from Philadelphia were compared to each other and polymorphisms were assessed (Table S4). Genomes differed from their nearest neighbor within this data set by 0 to 9 substitutions (mean = 1.9). In 14 of 27 of these cases, the match to another Philadelphia sequence was closer than the match to genomes from any other location. Analysis is complicated by incomplete sampling at all sites, but these observations are consistent with circulation of closely related lineages within the Philadelphia community.

Thus, the picture that emerges is that the first wave of the epidemic in Philadelphia was mostly introduced from New York, with additional less prominent introductions from a few other sites, and subsequently driven by spread due to circulation within the Philadelphia community. We attempted to specify the geographic origin of infection chains more precisely by analyzing possible clustering of the tree in Fig. 2 by subject zip code but did not find any significant clustering (permutational multivariate analysis of variance [PERMANOVA] *P* value of 0.6).

### Lack of association between viral polymorphisms and patient outcomes.

We next assessed possible associations of viral polymorphisms versus patient disease severity based on WHO scores for maximum severity reached (23) and outcomes. All patients studied (*n* = 27) were hospitalized (WHO score ≥4 [23]) and were grouped as moderate disease (nonintubated; maximum WHO score 4 to 6; *n* = 6), severe disease (intubated; WHO score 7 to 9; *n* = 13), or fatal outcomes (WHO score 10; *n* = 8) (Table 1 and Table S1). Polymorphisms were compared to severity (moderate/severe/fatal) and final outcomes (survivor/nonsurvivor) using PERMANOVA, which queries global associations of the sequence-based tree with outcome. No significant associations were found (*P* = 0.38 for WHO score and *P* = 0.06 for survival; prior to correction for multiple comparisons). Specific polymorphisms were next queried using Fisher’s exact test comparing polymorphisms versus outcomes. The closest to significance were a set of 4 single nucleotide polymorphisms (SNPs) (C18998T, C23230T, G29540A, and T1918C) that are shared between two patients (211 and 222) who both died (Fisher’s *P* value = 0.074; prior to correction for multiple comparison). Three of the polymorphisms are synonymous, while one caused A1844V in ORF1b. Thus, we did not detect viral polymorphisms that significantly increased or decreased pathogenic potential in our cohort, paralleling previous work (18).

### Longitudinal variation of viral sequences within subjects.

**FIG 4:**
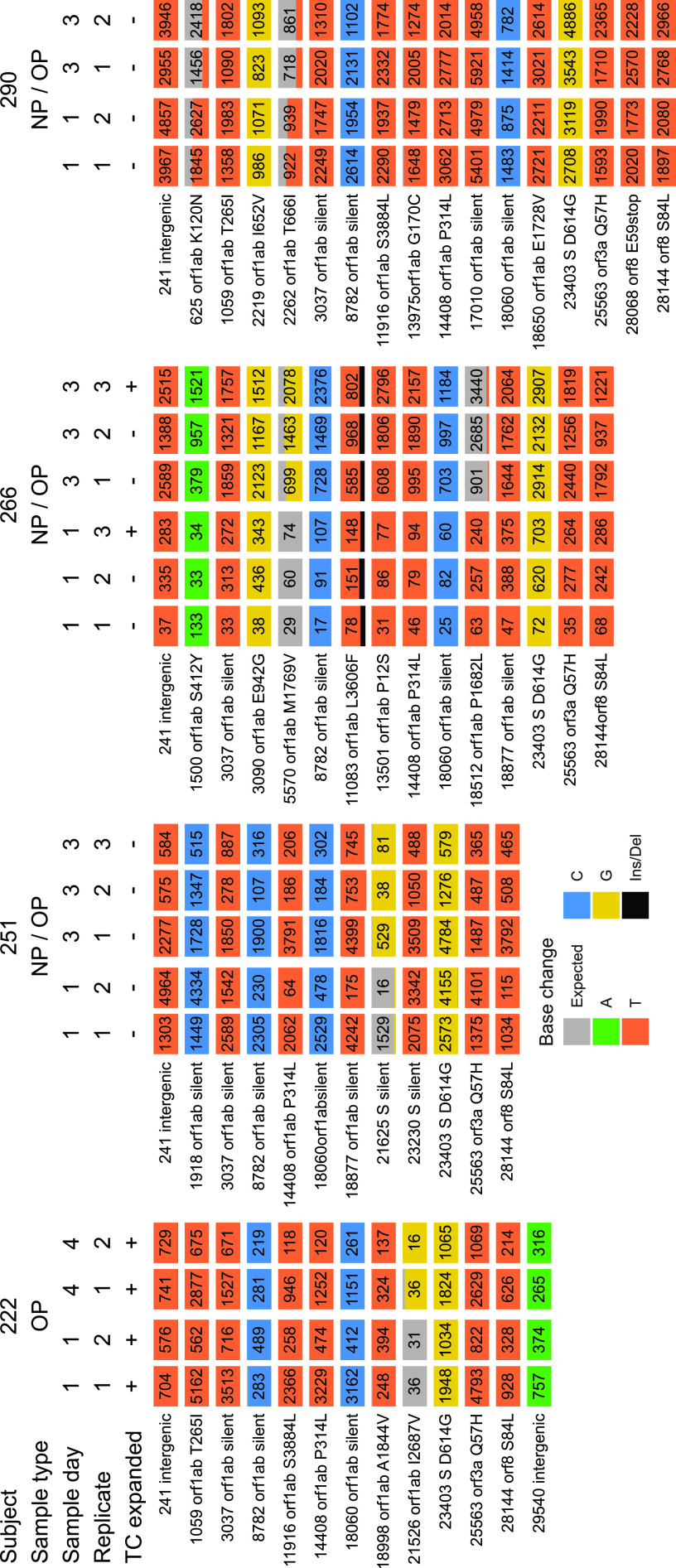
Examples of longitudinal variation detected in SARS-CoV-2 genome sequences. The plots show base substitutions differing from the USA-WA1-2020 reference isolate as colored tiles. Each column shows results of an independent determination of the genome sequence; the top of the column indicates (1) the subject of origin; (2) the sample type; (3) the relative days of sampling; (4) replicate number, i.e., indicating independent sequence determinations for the same patient sample; and (5) whether the virus was isolated and expanded in tissue culture (“TC expanded”). Results of technical replicates of the sequencing procedure are marked with numbers to indicate independent sequence determinations for the same initially isolated patient sample. The numbers of sequence reads contributing to the sequence call are marked on each tile. Tiles with more than one color indicate the presence of minor sequence variants at that position. The key to tile colors is at the bottom; gray indicates a match to the consensus USA-WA1-2020; Ins/Del indicates insertion/deletion.

We next investigated possible longitudinal variation (Fig. 4 and Table S5). We obtained high-quality complete genome sequences from the same body site at more than one time point for 8 patients. Intervals between samples ranged from 2 to 39 days. In the longest sampling period, subject 228 yielded identical viral genome sequences over 39 days. Identical genomes were recovered from NP, OP, NP-OP, and saliva samples over this period. Subject 228 is a 65-year-old male who reached WHO level 9 and was hospitalized for 55 days but ultimately survived to be discharged. This emphasizes that viral populations can show notable longitudinal stability in some subjects.

10.1128/mBio.03456-20.5 TABLE S5 Table of polymorphisms detected longitudinally at the same body site. Download Table S5, XLSX file, 0.01 MB. Copyright © 2021 Everett et al. 2021 Everett et al. This content is distributed under the terms of the Creative Commons Attribution 4.0 International license.

In four of eight cases, sequence data showed polymorphisms between time points. To verify the authenticity of polymorphisms, we repeated the sequencing procedure on an independent aliquot of the samples (Fig. 4, “replicate” designation), validating reproducible polymorphisms in all four cases. One polymorphism resulted in a silent substitution in the spike coding region, and the others resulted in amino acid coding changes in the large orf1ab gene. In all four cases, longitudinal variation was seen in upper respiratory tract samples, and not in the two ETA samples queried. In most cases, the variant present in the earlier samples was evident as a minor variant in the later samples, suggesting that standing viral populations commonly encode multiple variants at single loci and that proportions can change over time.

Either of two processes could account for the observed variation. The data are consistent with the idea that the virus is mutating within subjects, and rapid cell turnover and particle washout result in appearance of new variants. The alternative is that the subjects were infected initially with viral populations containing multiple variants, and different variants became predominant at different times during infection.

### Differences in viral genome sequences between body sites.

**FIG 5:**
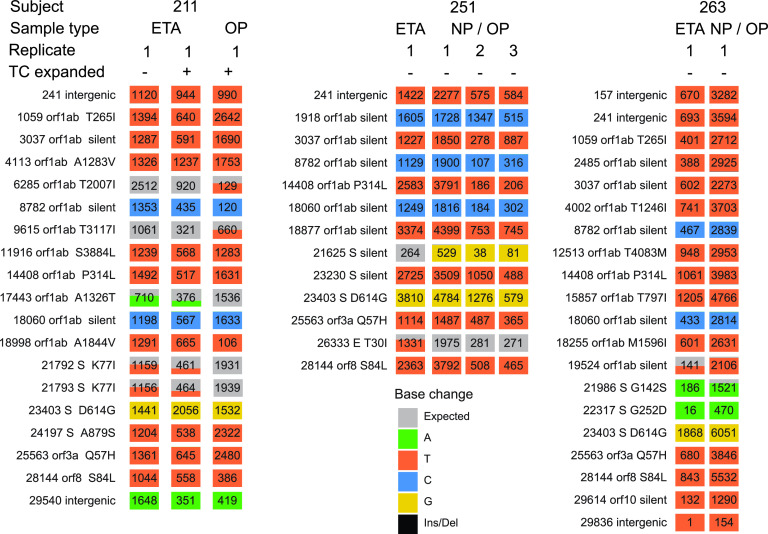
Examples of SARS-CoV-2 genome variation at different body sites at the same time of sampling. Markings are as in Fig. 4. The sample types are marked at the top of each column. Technical replicates of the sequencing procedure are shown separately.

Another question turns on whether there are separately evolving viral populations at different body sites. We were able to investigate 10 pairs of genomes from different body sites at the same time point (Table S6). Comparisons include different upper airway sites and upper versus lower airway. In 3 out of 10 cases, we identified polymorphisms differing at different body sites at the same time point (Fig. 5). In several cases, the polymorphic variant present in one of the sites was evident as a minor variant in the other site. In all three cases, the polymorphisms involved comparison of upper respiratory tract samples to an endotracheal aspirate. For the comparison of subject 211, there is a possible alternative explanation—the viruses compared were grown out first in tissue culture, so variants could have alternatively accumulated at this step. Nevertheless, these data together suggest possible independently replicating viral populations along the respiratory tract with incomplete intermixing in some subjects. As with the longitudinal data, variation could be the result of either *de novo* mutation within subjects or initial infection with multiple variants.

10.1128/mBio.03456-20.6 TABLE S6 Table of polymorphisms at different body sites within the same subject Download Table S6, XLSX file, 0.01 MB. Copyright © 2021 Everett et al. 2021 Everett et al. This content is distributed under the terms of the Creative Commons Attribution 4.0 International license.

### Variation associated with expansion in cell culture.

Nine of the viral isolates were expanded by growth in cell culture prior to sequencing, and for samples from two subjects (211 and 266), genomes were sequenced from patient samples before expansion as well. Samples were expanded by growth for 5 to 6 days on human A549 cells engineered to express the ACE2 viral receptor. Successfully expanded viral stocks were recovered from NP, OP, and ETA samples (Table S2).

For subject 211 (Fig. 5), five SNPs were identified that distinguish the tissue-culture expanded virus from the original ETA sample. Examination of the sequence reads showed that all five variants were detectable as minor populations that changed in proportion upon tissue culture, so that the majority form changed at the five loci. It is unknown whether this reflects superior replication of these variants under conditions of tissue culture or instead stochastics of sampling during the virus isolation procedure.

For subject 266 (Fig. 4), NP/OP samples from two dates were each expanded and compared to preexpansion sequences. In each case, the expanded sequence was identical to the preexpansion sequence.

### Possible consequences of the observed substitutions.

The data could be interrogated to investigate several additional aspects of viral evolution. The drug remdesivir is coming into wide use (24), and the binding site for remdesivir has been defined by structural analysis (25–27). Thus, we asked whether polymorphisms are accumulating in the binding site, suggestive of evolution to drug resistance. While only 3 of our subjects received remdesivir, such polymorphisms could indicate accumulation of resistant lineages circulating within the community sampled. However, no polymorphisms were detected in or near the region encoding the remdesivir binding site.

Koonin and coworkers identified regions of coronavirus genomes that they proposed to be associated with increased pathogenesis in humans, including regions encoding the spike protein and subcellular sorting motifs in the nucleoprotein (28). No polymorphisms were found in any of these sites, providing no support to the idea that SARS-CoV-2 is evolving to be more pathogenic in these subjects by these pathways.

The furin cleavage site in the spike protein has been proposed to be a locus of evolution in coronaviruses (29–32). However, no changes were observed in the region encoding the furin cleavage site in the genomes studied here.

ORF8 encodes a protein suggested to promote immune evasion by downregulating major histocompatibility complex (MHC) (33), and previously mutations in ORF8 were found and suggested to be associated with reduced severity of disease or altered immune evasion (34, 35). We found an example of a genome that in fact contained a stop codon in ORF8 (Fig. 4; subject 290). We resequenced this genome and verified that the substitution was indeed present. The possible consequences for replication are unknown; however, we note that subject 290 reached a WHO score of only 4 (hospitalized without supplemental oxygen) and survived to be discharged from the hospital, consistent with possible attenuation by the ORF8 substitution.

### Polymorphisms potentially disrupting SARS-CoV-2 detection.

Lastly, we checked whether commonly used reverse transcription-PCR (RT-PCR) and reverse transcription–loop-mediated isothermal amplification (RT-LAMP) primer sets for detecting SARS-CoV-2 (Table S7) were fully matched with Philadelphia isolates, or whether polymorphisms might disrupt viral detection (36, 37). For this, we compared the CDC RT-PCR primers and several widely used RT-LAMP primer sets (38, 39). No polymorphisms were found in binding sites for the CDC RT-PCR primers. Of the three RT-LAMP primer sets studied, each did have at least one primer for which binding would be disrupted by a patient polymorphism; in each case, the polymorphism was found in one subject only. Thus, we conclude that all of these primer sets are suitable for detecting the great majority of SARS-CoV-2 lineages in Philadelphia, but in rare instances sensitivity for some may be decreased by target site polymorphisms.

10.1128/mBio.03456-20.7 TABLE S7 Table of primers commonly used for detection and polymorphism mismatched in isolates from Philadelphia. Download Table S7, XLSX file, 0.01 MB. Copyright © 2021 Everett et al. 2021 Everett et al. This content is distributed under the terms of the Creative Commons Attribution 4.0 International license.

## DISCUSSION

Here, we present an analysis of the SARS-CoV-2 epidemic in Philadelphia using viral whole-genome sequencing of 52 isolates from 27 hospitalized patients. We find that all of the viral genomes recovered contained the spike D614G substitution suggested to promote spread among humans, and all contained the linked RdRp P314L substitution, marking them as the lineage B.1, Nextstrain clade 20A or 20C, GISAID clade G or GH, and clade A2a (16, 17). The majority of these genomes had nearest neighbors outside Philadelphia that were most commonly from New York, providing a probable origin for much of the outbreak in Philadelphia. In a few cases, genomes had best matches to genomes from other locations, consistent with additional independent introductions. When comparing sequences within Philadelphia, many were found to have even closer matches to local sequences, suggesting community transmission chains. We thus propose that the epidemic in Philadelphia was seeded primarily from New York, followed by local spread. Although power to detect differences was small, in no case did any polymorphism correlate with patient outcome, consistent with results of others (18).

Use of whole-genome sequencing to postulate transmission chains involves several approximations, so that conclusions must be taken as likely scenarios and not strictly established. When aligning the Philadelphia isolates to database genomes, it was common to find multiple equally good closest matches, and usually this collection contained isolates from several different locations. In the interpretation, we focused on the most frequently seen location, typically New York, but it is not ruled out that transmission could have been from one of the other less frequently captured locations with high-homology genomes. A bias in this analysis is that sampling effort is not distributed equally at different global sites, resulting in potentially more frequent identification of isolates from heavily sampled regions. In addition, the sharing of two sequences between different locations at different times is taken to suggest transmission from one location to the other, but both might have been infected independently from some third location. Our interpretation nevertheless seems warranted—the finding of nearest external neighbors in New York was the case for many of our genomes, and similarly the frequency of still more homologous within-Philadelphia best matches was also high. Thus, the data support a model of infection primarily from New York followed by local spread.

Our study recovered multiple complete genome sequences from individual patients at different times after infection, allowing assessment of within-host sequence heterogeneity. To exclude possible error in sequence determination, where possible we resequenced virus with possible polymorphisms from an independent sample aliquot to validate the variants detected. In four out of eight of the subjects analyzed, reproducible polymorphisms were detected. This finding implies either that populations of SARS-CoV-2 are accumulating substitutions and turning over at a high rate or else that patients were initially infected with multiple variants and different variants predominated at different times. High mutation rates are well known in many RNA viruses such as HIV and hepatitis C virus (HCV) (40).

We were also able to study 10 cases where high-quality genomes were available from different body sites at the same time in the same individual. Of these, three showed polymorphisms. Although the numbers are small, this suggests that there can be heterogeneity of viral populations at different body sites and limited exchange between sites. All three cases involved comparison of lung (endotracheal aspirate) samples to upper respiratory tract samples, potentially consistent with distinct viral populations replicating in the upper and lower respiratory sites in these patients. As with the analysis of longitudinal variation, it is unknown whether this reflects initial infection with a mixed virus population or accumulation of sequence variation during growth within subjects.

This study has several limitations. All subjects studied were hospitalized, leaving open the possibility that different genotypes predominate in less sick subjects. We analyzed small numbers of subjects with genomes in samples collected longitudinally or contemporaneously at multiple body sites—it will be valuable to examine more subjects to determine whether the extent of polymorphism seen here is reproducible in other cohorts. Happenstance of sampling may have also affected recovery of sequence polymorphism—for example, if different nostrils were sampled at different times, a temporal difference might be inferred that actually represented partitioning by body site. We note that these observations of potential differences in viral populations in time and space should be amenable to further investigation using experimental infections in model organisms.

In summary, our complete genome sequence analysis indicates that the SARS-CoV-2 epidemic in Philadelphia was primarily seeded from New York, which experienced an earlier expansion of COVID-19, followed by local spread. We were able to survey several subjects longitudinally, in some cases observing acquisition of viral polymorphisms that imply either initial infection with heterogeneous viral populations or accumulation of variants during growth within subjects. We also saw examples of polymorphisms in different body sites in some subjects, suggesting that populations may distribute in part independently at different anatomical locations. These observations present new hypotheses for viral dynamics that should be readily amenable to further study.

## MATERIALS AND METHODS

### Human subjects.

Following informed consent obtained under protocol no. 823392 approved by the University of Pennsylvania IRB, samples were collected beginning within 2 days of hospitalization. Oropharyngeal (OP) and nasopharyngeal (NP) swabs were obtained using flocked swabs (Copan Diagnostics) eluted in 1.5 to 3 ml of viral transport medium. In some instances, OP and NP samples were eluted together (NP-OP). Saliva and endotracheal aspirate samples were obtained from nonintubated or intubated patients, respectively. Patients were classified clinically based on survival to discharge or in-hospital mortality and on the maximum score reached during hospitalization based on the 11-point WHO clinical COVID-19 progression scale (23).

### RT-qPCR to detect SARS-CoV-2.

RNA was extracted from 140 µl of clinical sample (swab eluate or neat endotracheal aspirate or saliva) using the Qiagen QIAamp viral RNA minikit. The RT-quantitative PCR (qPCR) assay targeted the SARS-CoV-2 nucleocapsid region using the CDC 2019-nCoV_N1 primer-probe set (2019-nCoV_N1-F, GACCCCAAAATCAGCGAAAT; 2019-nCoV_N1-R, TCTGGTTACTGCCAGTTGAATCTG; 2019_nCoV_N1-P, 6-carboxyfluorescein (FAM)-ACCCCGCATTACGTTTGGTGGACC-Iowa black fluorescent quencher (IBFQ). The RT-qPCR master mix was prepared according to the following protocol: 8.5 µl distilled water (dH_2_O), 0.5 µl N1-F (20 µM), 0.5 µl N1-R (20 µM), 0.5 µl N1-P (5 µM), 5.0 µl TaqMan Fast Virus 1-Step master mix per reaction. Five microliters of extracted RNA was added to 15 µl of prepared master mix for a final volume of 20 µl per reaction. Final concentrations of both 2019-nCoV_N1-F and 2019-nCoV_N1-R primers were 500 nM, and the final concentration of the 2019-nCoV_N1-P probe was 125 nM as suggested by the CDC protocol. The assay was performed using the Applied Biosystems QuantStudio 5 real-time PCR system. The thermocycler conditions were as follows: 5 min at 50°C, 20 s at 95°C, and 40 cycles of 3 s at 95°C and 30 s at 60°C.

### Cells.

Human A549 cells expressing ACE2, constructed by lentivirus transduction of *hACE2* (41), were cultured in RPMI 1640 (Gibco catalog no. 11875) supplemented with 10% fetal bovine serum (FBS), 100 U/ml of penicillin, and 100 μg/ml streptomycin.

### Viral isolation.

Nine viral isolates from patients were expanded in cell culture prior to sequencing. Successfully expanded viral stocks were recovered from NP, OP, and ETA samples. Briefly, NP or OP swabs were incubated with 1 ml of viral isolation medium (DMEM [Gibco catalog no. 11965] with 200 U penicillin and 200 μg/ml streptomycin) for 1 h at room temperature. For ETA samples, 500 µl or 100 µl of eluate was mixed with 500 or 900 µl viral isolation medium and then inoculated onto A549^ACE2^ cells in 48-well plates. After 1 h of inoculation, the media were removed from the wells, and 1 ml of culture medium (RPMI 1640 [Gibco catalog no. 11875] with 2% FBS and 200 U penicillin and 200 μg/ml streptomycin) was added to each well. Three to 4 days postinfection, supernatants were harvested and 300 µl was used to inoculate A549^ACE2^ cells in 6-well plates. Forty-eight hours postinfection, the supernatants were collected, the cells were lysed using RLT Plus lysis buffer, and the RNA was extracted using the RNeasy Plus minikit (Qiagen). For viral isolates, please contact Susan Weiss.

### Viral genome sequence acquisition.

Most viral genome sequencing was carried out using the POLAR protocol (19). For each sample, 1 μl to 5 μl of viral RNA was used along with 0.5 μl of 10 mM deoxynucleoside triphosphate (dNTP) mix (Thermo Fisher, 18427013), 0.5 μl of 50 μM random hexamers (Thermo Fisher, N8080127), and additional nuclease-free water to reach a total volume of 6.5 μl. The mixture was incubated at 65°C for 5 min followed by a 1-min incubation at 4°C. Reverse transcription was performed by the addition of 0.5 μl SuperScript III reverse transcriptase (Thermo Fisher, 18080085), 2 μl of 5× First-Strand buffer (Thermo Fisher, 18080085), 0.5 μl of RNaseOUT (Thermo Fisher, 18080051), and 0.5 μl of 0.1 M dithiothreitol (DTT) (Thermo Fisher, 18080085). The reverse transcription mixture was incubated at 42°C for 50 min and 70°C for 10 min and then held at 4°C. To amplify the cDNA, we used the artic-ncov2019 version 3 primers designed by the ARTIC Network. The two pooled ARTIC primer sets were provided by IDT. For the PCR, 5 μl of 5× Q5 reaction buffer (NEB, M0493S), 0.5 μl of 10 mM dNTP mix (NEB, N0447S), 0.25 μl Q5 Hot Start DNA polymerase (NEB, M0493S), and either 4.0 μl of pooled primer set 1 or 3.98 μl of pooled primer set 2 were prepared for each reaction. Additionally, 12.7 μl or 12.8 μl of nuclease-free water and 2.5 μl of cDNA were added to reach a total volume of 25 μl. The reaction mixture was incubated at 98°C for 30 s for 1 cycle, followed by 25 cycles at 98°C for 15 s and 65°C for 5 min, and then held at 4°C. Prior to using the ARTIC nCoV-2019 amplicon sequencing protocol, we designed 12 primers that amplified six regions along the SARS-CoV-2 genome. In these early experiments, the random hexamers and ARTIC primers were replaced with our genome-specific primers.

PCR products of the same genome that were generated from the two primer sets were pooled. A 1:1 volume AMPure XP (Beckman Coulter, A63881) bead cleanup using 80% ethanol washes and 15 μl elution in nuclease-free water was carried out before quantifying the DNA content with the Qubit dsDNA HS assay kit (Thermo Fisher, Q32851). PCR products were diluted to 0.25 ng/μl, and the Nextera library was prepared using the Nextera XT library prep kit (kit (Illumina, FC-131-1096) and the Nextera XT Index kit (Illumina, FC-131-2001, FC-131-2002). The DNA tagmentation reaction, adaptor ligation, and amplification for the library followed the protocols in the Nextera XT library prep reference guide provided by Illumina. Following the Nextera amplification, a second 1:1 volume AMPure XP bead cleanup was done, and samples were eluted in 35 μl of nuclease-free water. RT-PCR was used to quantify the DNA of each sample using the KAPA SYBR Fast Universal qPCR kit (Roche, KK4903, KK4622). The samples were pooled in equal quantities, and an additional RT-PCR was performed on the pooled library. The library was sequenced on an Illumina MiSeq.

### Genome analysis.

A custom informatics pipeline was created to align quality trimmed reads to the USA-WA1-2020 reference genome with the Burrows-Wheeler aligner and create read pile-ups and call variants with the SAMtools/bcftools software packages (42–45). Variants were accepted if they possessed a PHRED score of  ≥20, the position had a read depth of at least 5 reads, and >50% of the reads included the variant. The pipeline created data objects for each sequenced sample and subsequently compiled these data objects into detailed subject reports including variant heatmaps and read pileups for both individual experiments and compilations of multiple experiments. One genome sequence was excluded because repeated attempts at sequence acquisition failed to yield a consensus sequence. Genomes from another subject were excluded when parallel analysis of HLA-A and HLA-B RNA failed to confirm that samples were from the same subject. The phylogenetic tree of local genomes shown in Fig. 2 was created via hierarchical cluster analysis using the UPGMA (unweighted pair group method with arithmetic mean) algorithm (46) where global genome alignments were first created with the MAFFT alignment software package (47). Maximum-likelihood phylogenies of global genome representatives shown in Fig. 3 (sampling of PANGOLIN lineages) were built with the IQ-TREE software package (48), implemented through the Augur informatics package (49), which employs a combination of hill-climbing and stochastic perturbation methods to build trees from large data sets.

### Data availability.

All sequence data acquired in this study are available at NCBI under MW001232 to MW001286; Table S2 in the supplemental material relates the sample metadata to sequence accession numbers. Computer code used is available at https://doi.org/10.5281/zenodo.4046252. A detailed list of materials used is in Table S8.

10.1128/mBio.03456-20.8 TABLE S8 Table of materials used in this study. Download Table S8, XLSX file, 0.01 MB. Copyright © 2021 Everett et al. 2021 Everett et al. This content is distributed under the terms of the Creative Commons Attribution 4.0 International license.
